# Reduced functional connectivity of fronto-parietal sustained attention networks in severe childhood abuse

**DOI:** 10.1371/journal.pone.0188744

**Published:** 2017-11-30

**Authors:** Heledd Hart, Lena Lim, Mitul A. Mehta, Antonia Chatzieffraimidou, Charles Curtis, Xiaohui Xu, Gerome Breen, Andrew Simmons, Kah Mirza, Katya Rubia

**Affiliations:** 1 Department of Child & Adolescent Psychiatry, Institute of Psychiatry, Psychology and Neuroscience, King’s College London, London, United Kingdom; 2 Department of Neuroimaging, Institute of Psychiatry, Psychology and Neuroscience, King’s College London, London, United Kingdom; 3 MRC SGDP Centre, NIHR BRC for Mental Health, Institute of Psychiatry, Psychology & Neuroscience & SLaM NHS Trust, King's College London, London, United Kingdom; Stellenbosch University, SOUTH AFRICA

## Abstract

Childhood maltreatment is associated with attention deficits. We examined the effect of childhood abuse and abuse-by-gene (*5-HTTLPR*, *MAOA*, *FKBP5*) interaction on functional brain connectivity during sustained attention in medication/drug-free adolescents. Functional connectivity was compared, using generalised psychophysiological interaction (gPPI) analysis of functional magnetic resonance imaging (fMRI) data, between 21 age-and gender-matched adolescents exposed to severe childhood abuse and 27 healthy controls, while they performed a parametrically modulated vigilance task requiring target detection with a progressively increasing load of sustained attention. Behaviourally, participants exposed to childhood abuse had increased omission errors compared to healthy controls. During the most challenging attention condition abused participants relative to controls exhibited reduced connectivity, with a left-hemispheric bias, in typical fronto-parietal attention networks, including dorsolateral, rostromedial and inferior prefrontal and inferior parietal regions. Abuse-related connectivity abnormalities were exacerbated in individuals homozygous for the risky C-allele of the single nucleotide polymorphism rs3800373 of the FK506 Binding Protein 5 (FKBP5) gene. Findings suggest that childhood abuse is associated with decreased functional connectivity in fronto-parietal attention networks and that the *FKBP5* genotype moderates neurobiological vulnerability to abuse. These findings represent a first step towards the delineation of abuse-related neurofunctional connectivity abnormalities, which hopefully will facilitate the development of specific treatment strategies for victims of childhood maltreatment.

## Introduction

Child abuse is, regrettably, common with twenty-two percent of adolescents in the UK reporting lifetime physical, emotional, sexual abuse or neglect [1]. Childhood maltreatment causes extreme stress which, via physiological, neurochemical, and hormonal changes, can lead to alterations in brain structure, function and connectivity most consistently in fronto-limbic areas and networks [2, 3], but with some evidence for alterations also in temporal and parietal regions [4–6].

Neuropsychological studies of childhood maltreatment have reported auditory [7, 8] and visual [8–12] attention deficits. Sustained attention, the ability to keep one's mind continuously focused on a particular task, is a key dimension of attention control [13] and is important for mature goal-directed behavior, thought to underpin higher-level attention processes such as selective and divided attention as well as general cognitive ability [14]. Children with maltreatment-related PTSD [9] and institutionalized children make more omission errors than healthy controls during sustained attention, which are related to longer institutional care [15, 16]. Sustained attention deficits have also been reported in adults with childhood physical abuse and neglect histories [17].

Despite consistent neuropsychological findings of attention deficits in maltreated children, to date only one functional magnetic resonance imaging (fMRI) study has examined sustained attention in individuals exposed to childhood maltreatment. Previously published data by our group suggest that childhood abuse is associated with reduced activation during the most challenging attention condition in the same sustained attention task used in the current study, compared to healthy controls, in typical dorsal and ventral attention networks including left dorsolateral prefrontal cortex (DLPFC), inferior frontal cortex (IFC) and temporal regions [18]. Other fMRI studies in childhood maltreatment have reported alterations in activation during emotion processing [19–21]; motor response inhibition [22–24] and working memory [25].

Most fMRI studies of childhood maltreatment have concentrated exclusively on functional activation and neglected more sophisticated functional connectivity analyses. Functional communication between brain regions is vital in cognition and the few published functional connectivity studies of child abuse demonstrated altered connectivity of diffuse neural networks, fronto-limbic in particular, during resting state [26, 27], emotion processing [21, 28, 29] and response inhibition [30]. These preliminary findings suggest that it is crucial to better understand the effect of maltreatment on brain networks in addition to isolated regions. This is of particular relevance as childhood trauma has been shown to affect the morphometry and integrity of white matter tracts [31–33] and functional connectivity strength has been shown to correlate with structural connectivity of white matter tracts in the same regions [34].

Although childhood maltreatment is an important risk factor for several psychiatric disorders, it does not invariably lead to dysfunction. It is recognized that genetic differences influence the likelihood that abuse exposure will result in psychopathology [35] so it is important to examine if the abuse-related brain abnormalities are sensitive to gene-by-environment (GxE) interactions. GxE studies on early stress including childhood maltreatment show increased risk for emotional and antisocial behavioural problems in youth with the long (L) allele of the 5-HTTLPR polymorphism of the serotonin transporter gene [36–40] and the low activity variant of the variable number tandem repeat (*VNTR*) polymorphism of the monoamine oxidase type A (*MAOA*) gene [41–45]. Risk alleles of four common single nucleotide polymorphisms (SNPs) (rs1360780, rs3800373, rs9470080, rs9296158) of the FK506-binding protein 51 (FKBP5), which regulates glucocorticoid receptor sensitivity, have been reported to interact with childhood trauma to predict PTSD symptomatology [46], limbic irritability, depression and dissociation [47].

This study therefore examined the association between severe childhood maltreatment and functional connectivity of sustained attention networks in medication-naïve, drug-free young people using a parametrically modulated vigilance task requiring target detection with a progressively increasing load of sustained attention. As, during a previous study of the same sample [18], functional activation group differences were found only for the most challenging attention condition, the current paper focuses on functional connectivity for this condition only. As different forms of abuse present differently clinically (e.g., [48]) and likely have different effects on behaviour and neurobiology effects of different maltreatment types should ideally be considered separately. The current study aimed to investigate the effect specific to physical child abuse on the brain. Sexual abuse was excluded due to the known differences in structural, behavioural and psychiatric consequences [48, 49]. Preferably we would also have excluded neglect and emotional abuse but this was not possible as all cases of physical abuse that we identified had also experienced some degree of emotional abuse and/or neglect. This is representative of the abused population as most forms of child abuse do not occur in isolation [50, 51]. Based on evidence of the role of fronto-parieto-temporal regions in sustained attention [52–57], altered structure and function of these regions in individuals with a history of childhood maltreatment [2–4, 22, 25], the fact that they have been shown to develop relatively late in childhood and be progressively more activated with increasing age between childhood and adulthood [53, 58], and in particular our previous findings of decreased activation of dorsal and ventral fronto-temporal sustained attention regions [18], we hypothesized that the abused group, relative to healthy controls, would have abnormal functional connectivity of dorsolateral and inferior fronto-parieto-temporal networks during sustained attention. We also explored if these abnormalities would be moderated by *5-HTTLPR*, *MAOA* or *FKBP5* polymorphisms.

## Materials and methods

### Participants

Fifty (23 maltreated and 27 healthy controls) right-handed, medication-naïve, drug-free and age-matched youths between the ages of 13 and 20 years old were initially assessed using the Development and Well-Being Assessment (DAWBA) [59]. The Strengths and Difficulties Questionnaires (SDQ) [60] and Beck’s Depression Inventory (BDI) [61] were used to provide psychopathology symptom scores. IQ was assessed using the Wechsler Abbreviated Scale of Intelligence (WASI) [62]. The Childhood Trauma Questionnaire (CTQ) [63] was used to measure the severity of childhood physical, emotional and sexual abuse and emotional and physical neglect. Socioeconomic status (SES) was measured by two items from the Family Affluence Scale (FAS) [64] on housing tenure and room occupancy.

A 10 panel T-cup urine test (http://www.testfield.co.uk) was used to test for substance abuse. Participants who tested positive for any of the 10 substances were excluded resulting in the exclusion of 4 participants (3 maltreated and 1 healthy control). Other exclusion criteria were left-handedness, IQ < 70, current psychoactive medication, sexual abuse (as defined by a score of ≥ 6 on the sexual abuse subscale of the CTQ), neurological disorder, major head injuries, drug and alcohol abuse, literacy problems, learning disability, psychotic illness, bipolar disorder, schizophrenia, current suicidal behaviour or general MRI contraindications. Participants received £40 to compensate for their time and travel. The National Research Ethics Service reviewed and approved the study and informed written consent was obtained from all participants and, if below 18 years old, informed written consent was also obtained from parents or guardians. Participants were recruited and scanned during the period 2011 to 2013.

Twenty-three physically maltreated participants were recruited through Kids Company (http://www.kidsco.org.uk/), Child and Adolescent Mental Health Services (CAMHS) and advertisements. They scored ≥ 13 (i.e. the cut-off for severe/extreme physical abuse) on the CTQ physical abuse subscale and the abuse history was corroborated by social service records and the Childhood Experience of Care and Abuse (CECA) interview [65]. Head motion is a well-known confound of both resting state functional connectivity [66, 67] and task based fMRI data [68]. In order to reduce the likelihood of false positives caused by head movement we therefore excluded participants with root mean square (RMS) realignment estimates exceeding 1 mm. This was calculated from realignment parameters (rotational estimates converted to translational at radius of 50 mm) as described by Siegel et al. [68] and resulted in the exclusion of two maltreated participants, leaving a final sample of 21.

The 27 healthy controls with no history of psychiatric illness and childhood maltreatment (scoring below the same cut-offs as above) were recruited through advertisements in the same geographic areas of South London to ensure similar socioeconomic background (Table 1). All healthy controls had RMS movement < 1mm.

**Table 1:** Demographic characteristics of 21 young people exposed to severe childhood abuse and 27 healthy controls (CA = childhood abuse; HC = healthy controls; ADHD = attention deficit hyperactivity disorder; PTSD = post-traumatic stress disorder; ODD = oppositional defiant disorder; CD = conduct disorder).

	Childhood Abuse		Healthy Controls	
	(N = 21)		(N = 27)	
	Mean	SD	Mean	SD
**Age (years)**	17.5	2.32	17.5	1.63
**[Age Range: 13–20]** **Socioeconomic status**	2.77	0.69	3.22	0.75
**IQ**	90	12.6	105.4	10.1
**Strengths and Difficulties Questionnaire:**					
*Emotional problems*	4.62	2.77	1.92	1.61
*Conduct problems*	4.43	2.01	1.68	1.6
*Hyperactivity*	5.38	2.4	2.84	2.14
*Peer problems*	3.81	1.54	1.16	1.72
*Prosocial*	7.24	1.7	8.08	1.41
*Total difficulties score*	18.2	6.2	7.6	5.73
**Beck’s Depression Inventory**	16	10.6	5.92	6.09
**Childhood Trauma Questionnaire:**					
*Physical abuse*	20.8	5.04	5.52	0.94
*Emotional abuse*	18	4.4	6.04	1.13
*Sexual abuse*	5.14	0.65	5.11	0.42
*Physical neglect*	14	5.02	5.59	1.22
*Emotional neglect*	18.3	3.93	7.93	3.35
**Age at onset of abuse (years)**	4.24	2.55		
**Duration of abuse (years)**	8.29	3.2		
	**N**	**%**	**N**	**%**
**Gender (Males)**	15	71	21	77
**Ethnicity:**				
*White*	10	48	13	48
*Afro-Caribbean*	8	38	12	44
*Others (Asian/mixed)*	3	14	2	8
**Psychiatric diagnosis:**				
*PTSD*	12	57	-	
*Depression*	6	29	-	
*Anxiety disorders*	4	19	-	
*Social phobia*	1	5	-	
*ADHD*	1	5		
*ODD/CD/Other disruptive behaviors*	4	19		

### Genotyping

Genotyping of the *5-HTTLPR* promoter region polymorphism, the *MAOA* 30 bp-promoter and four common SNPs (*rs1360780*, *rs3800373*, *rs9470080*, *rs9296158*) of FKBP5 were carried out using previously described methods [69–71]. Individuals were identified as risk allele carriers or not: i.e., long for *5-HTTLPR*, short/low for *MAOA*, T-allele carriers for *rs136078* and *rs94700800*, A-allele carriers for *rs9296158 and* C-allele carriers for *rs3800373*.

### fMRI paradigm: Sustained attention task (SAT)

Participants practiced the task once prior to scanning. The 12-min SAT is a variant of psychomotor vigilance and delay tasks [53, 54]. Participants need to respond as quickly as possible to the appearance of a visual timer counting up in milliseconds via a right hand button response within 1s. The visual stimuli appear either after short, predictable consecutive delays of 0.5s, in series of 3–5 stimuli (260 in total), or after unpredictable time delays of 2s, 5s or 8s (20 each), pseudo-randomly interspersed into the blocks of 3–5 0.5s delays. The long, infrequent, unpredictable delays place a higher load on sustained attention/vigilance while the short, predictable 0.5s delays are typically anticipated [72] placing a higher demand on sensorimotor synchronization [53, 54, 73] (S1 Fig).

### Performance data analysis

Independent sample t-tests were used to compare the main variables of the sustained attention task performance between the abused and the control group using SPSS 21: mean reaction time (RT), intrasubject standard deviation of mean RT (SDintrasubject), omission and premature errors. T-tests for the short delays (0.5s) were also conducted separately on the same measures.

### fMRI image acquisition and analysis

Details of image acquisition, preprocessing and first and second-level functional activation analyses methods and results are published elsewhere [18].

### Functional connectivity analysis

As functional group differences were found only for the 8s delay condition [18], the current paper focuses on functional connectivity group differences for this condition only by conduction of a generalised psychophysiological interaction (gPPI) analysis using SPM8. Ten seed regions were selected: 1,2) Left and right anterior insula (-38,26,16; +38,26,16); 3,4) Left and right dorsolateral prefrontal cortices (DLPFC) (-35,35,39; +37,37,38); 5,6) Left and right inferior frontal cortices (IFC) (-47,31,13; +49,31,13); 7,8) Left and right inferior parietal lobes (IPL) (-41,-47,48; +45,-46,48); 9,10) Left and right superior temporal gyri (STG) (-48, -14,2; +54, -8, -2). These seed regions were chosen based on independent data from previous studies which have demonstrated consistent evidence for their involvement in sustained attention [55, 74–79], in the current task in particular [53, 54, 56] and are brain regions which have also been implicated in previous studies of childhood maltreatment (For a review see [2]). Co-ordinates for all seed regions were selected as the centroids of the region of interest (ROI) as defined using wfupickatlas [80] and aal [81]. For each seed region, at the individual subject level, an average time course was extracted defined as an 8 mm sphere around the abovementioned coordinates for use in the gPPI analysis.

The gPPI toolbox (http://www.nitrc.org/projects/gppi) was used to investigate the interaction effect during our contrast of interest (8s delay vs 0.5s implicit baseline) for all 10 seed regions. The deconvolved time series from the seed region was extracted for each participant to create the physiological variable and condition onset times were separately convolved with the canonical haemodynamic response function for each condition, creating the psychological regressors. Interaction terms (gPPIs) were computed by multiplying physiological and psychological variables and activity within the seed region was regressed on a voxel wise basis against the interaction, with the physiological and psychological variables serving as regressors of interest. Individual gPPI contrast images were entered into separate second level analyses to compare groups. Thus, the resulting activation maps from this analysis correspond to group differences for functional connectivity between the seed region and other brain regions during sustained attention. Results are reported using a cluster threshold of p < 0.05 family-wise error rate (FWER) corrected. Given the limited studies testing brain function differences in physically abused populations, and to control for the false positive rate (using p<0.05 family-wise error rate-corrected cluster statistics) while limiting potential type II errors, we chose an a priori cluster-forming threshold of P<0.001 for significant between-group differences.

Finally, significant clusters were extracted for exploratory correlational analysis with the performance measures for the 8s delay condition within both groups (mean RT, SDintrasubject, omission errors, premature errors) and abuse measures within the maltreated group only (onset, duration, CTQ score). Preliminary analysis of GxE effect on the significant clusters was conducted using ANOVAs with group and genotype (*5-HTTLPR*, *MAOA*, *rs1360780*, *rs3800373*, *rs9470080*, *rs9296158*) as between-subject factors.

## Results

### Subject characteristics

Groups did not differ significantly on age (t(46) = 0.03, p = 0.97), gender (t(46) = 0.08, p = 0.94), ethnicity (t(47) = 0.48, p = 0.51) nor socioeconomic status (t(47) = 1.49, p = 0.14) but differed on IQ as expected (t(47) = 4.70, p<0.001) (Table 1). Since lower IQ is associated with childhood maltreatment [11], artificially matching groups on IQ is inappropriate as it creates unrepresentative groups; either the abused group will have higher IQs than the abused population or the control group will have IQs below normative expectations [82]. Also, it is misguided to control for IQ differences by covarying IQ when groups are not randomly selected and the covariate is a pre-existing group difference as ANCOVA would lead to potentially spurious results [82, 83]. The primary data are therefore presented without matching or covarying IQ. However, to explore and rule out any potential influence of IQ, an analysis of covariance (ANCOVA) covarying for IQ was conducted.

Although we selected participants with severe childhood physical abuse, they also experienced marked/severe childhood emotional abuse and neglect (Table 1) which typically co-occur with physical abuse, and hence are a representative group of the abused population [50, 51]. Healthy controls scored significantly lower on BDI (*p* < 0.01) and all SDQ difficulties subscales (*p* < 0.001) than the abused group (Table 1).

### Task performance

Mean performance values are reported in Table 2. There was no significant group effect on mean reaction time (t(46) = 1.03; *p* = 0.31) but there was a significant group effect on intrasubject variability of mean reaction times (t(46) = 3.57, p < 0.001), with the maltreated group having greater intrasubject variability for all long delay conditions. There was also a significant group effect on omission (t(46) = 2.55, p < 0.05) and premature errors (t(46) = 2.58, p < 0.05), due to the abused group making more omission and premature errors than healthy controls (Table 2).

**Table 2:** Performance measures for the sustained attention task during 2s, 5s and 8s delays for 21 abused young people and 27 healthy controls. MRT = mean reaction time (in ms); SDintrasubject = intrasubject variability of mean reaction times (in ms); corr = Bonferroni corrected; CA = childhood abuse; HC = healthy control.

		Childhood Abuse (N = 21)		Healthy Controls (N = 27)	
	Delay	Mean	SD	Mean	SD
MRT	2s	446	64	411	59
	5s	450	78	414	74
	8s	449	87	408	80
SDintrasubject	2s	101	50	74	38
	5s	93	50	85	61
	8s	84	43	77	43
Omission errors	2s	0.33	0.73	0.11	0.42
	5s	0.57	0.93	0.19	0.48
	8s	0.62	1.2	0.04	0.19
Premature errors	2s	6.43	3.93	4	3.16
	5s	7.38	4.65	4.3	3.74
	8s	6.95	4.23	5.15	3.92

### Brain activation

#### Movement

Multivariate analyses of variance (MANOVAs) showed no significant group effects in the extent of 3-dimensional motion as measured by maximum displacement for x, y, and z axes (F(3,44) = 1.67; p = 0.14).

#### Functional activation

Within and between group functional brain activation is reported elsewhere [18]. Maltreated participants, relative to healthy controls, displayed significantly reduced activation during the most challenging attention condition only in typical dorsal and ventral attention networks including left dorsolateral and inferior prefrontal and temporal areas (Peak MNI coordinates: -38,26,16; -40,-54,-14). This was due to a significant linear trend of decreasing activation with increasing attention load in these regions in the abused group.

### Functional connectivity

#### Within group connectivity maps

S2 Fig shows within group functional connectivity maps for the different seed regions for the 8s delay vs the 0.5s implicit baseline.

#### Between group functional connectivity differences

**Fig 1:**
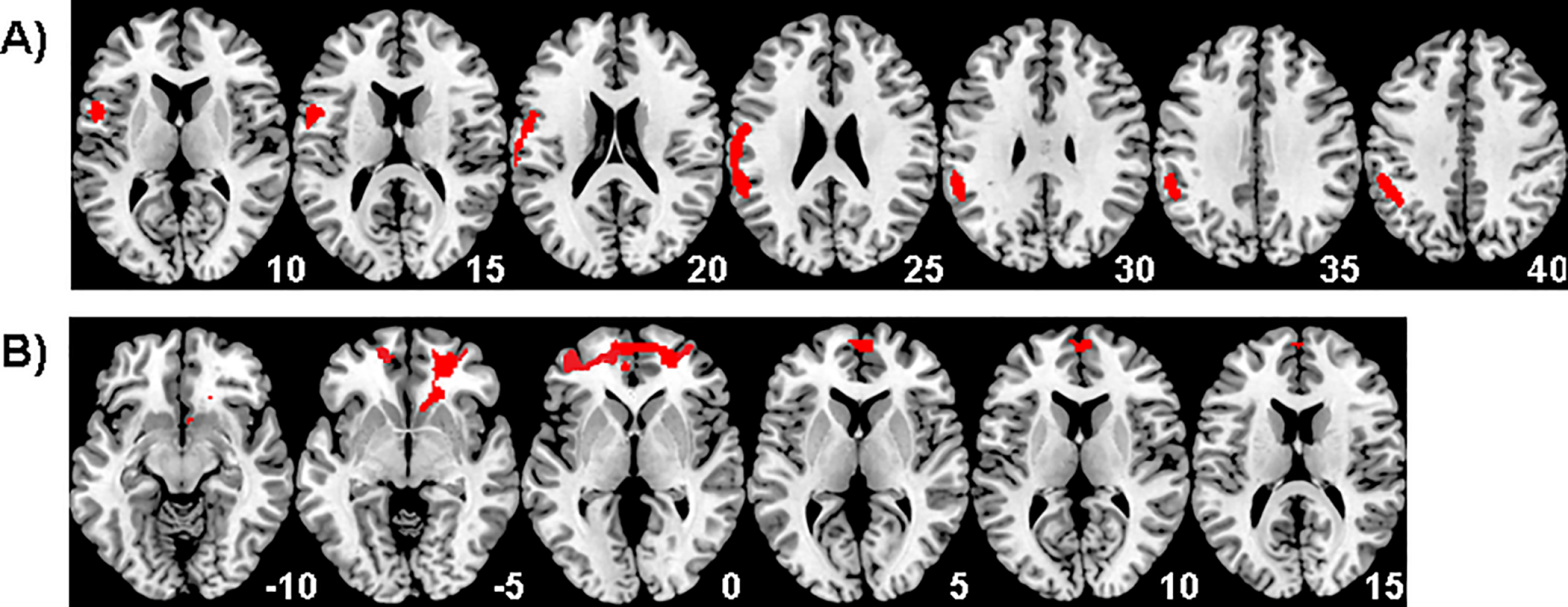
Functional connectivity differences between 21 physically maltreated young people and 27 healthy controls for the 8s delay condition vs 0.5s baseline. Illustrating regions that demonstrated reduced connectivity for maltreated participants compared to healthy controls with A) the seed region of the left dorsolateral prefrontal cortex and B) the left inferior parietal seed region. The threshold is P < 0.05 FWE corrected at the cluster level. Z-coordinates represent distance from the anterior–posterior commissure in mm. The right side of the image corresponds to the right side of the brain.

A significant reduction in connectivity in the abused group relative to healthy controls was revealed for the left DLPFC seed region with left IPL, supramarginal gyrus, IFC, postcentral and precentral gyri (BA 40/44/3/6) during the 8s delay condition (F(1,46) = 16.91; p<0.001), (Table 3, Fig 1).

**Table 3:** Regions demonstrating differential functional connectivity with the left dorsolateral prefrontal cortex and left inferior parietal lobe seed regions during the 8s delay versus 0.5s implicit baseline condition for 21 young people exposed to severe childhood abuse and 27 healthy controls. P-value is <0.05 FWER corrected.

		Cluster Level		Peak	Voxel Level
Seed Region	Comparison and Brain Regions	No. of Voxels	*p (corr)*	MNI Coordinates	Z
**L DLPFC**	**Physically Maltreated < Healthy Controls**				
	Left inferior parietal lobe, supramarginal gyrus, pars opercularis, inferior frontal, postcentral gyrus, precentral gyrus (BA 40/44/3/6)	730	0.012	-56,-42,30	4.14
**L IPL**	**Physically Maltreated < Healthy Controls**				
	Bilateral dorsolateral and rostromedial prefrontal cortex (BA 46/10)	687	0.032	-8,58,-4	4.02

For the left IPL seed region, a significant group effect for functional connectivity was shown with bilateral DLPFC and rostromedial prefrontal cortex (rmPFC) (BA 46/10) (F(1,46) = 14.55; p<0.001), which was due to reduced connectivity for maltreated compared to healthy adolescents (Table 3, Fig 1). No effect of group was observed for the remaining 8 seed regions.

### Exploratory analyses

#### Correlational analysis

No significant correlations were found between connectivity and performance or abuse measures.

#### IQ ANCOVA analysis

Given that the maltreated group had a significantly lower mean IQ than the healthy comparison group, data were reanalysed covarying for IQ. All main findings remained significant (S1 Table).

#### GxE analysis

**Fig 2:**
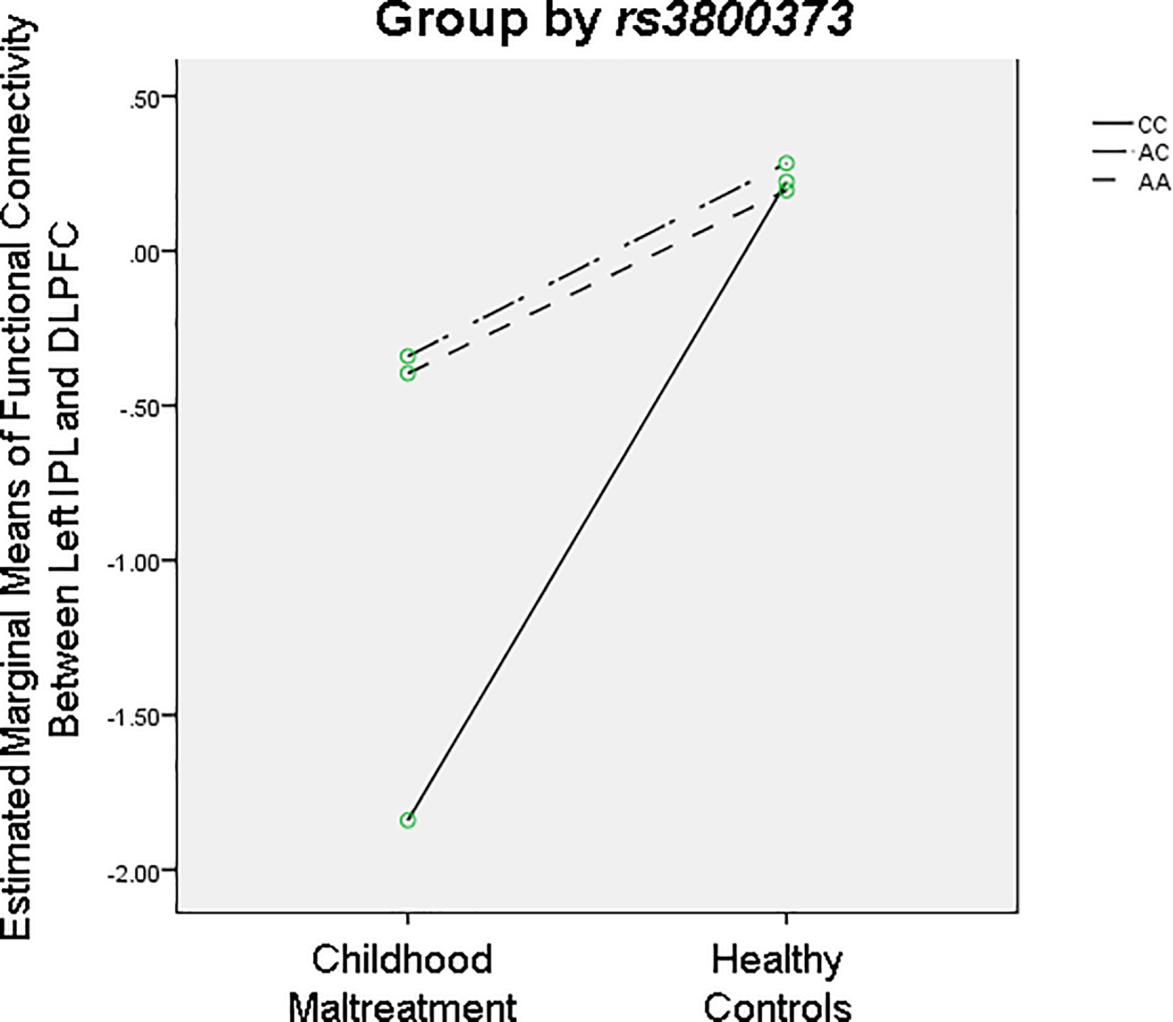
Significant GxE interaction effect between group (childhood abuse vs. healthy controls) and rs3800373 genotype (CC vs AC/AA) on functional connectivity between left IPL and DLPFC, p < 0.05.

Exploratory GxE analysis was conducted on the brain regions that differed in connectivity between the maltreated and healthy adolescents. ANOVAs with group (maltreated vs. healthy controls) and each genotype as between-subject factors showed a significant group-by-rs3800373 effect on connectivity between left IPL and left DLPFC (F (1,44) = 5.50, p < 0.05), due to a greater deficit in C-allele homozygotes exposed to abuse than A-allele carriers (Fig 2).

No significant group-by-genotype effects were observed for 5-HTTLPR, MAOA, rs1360780, rs9470080, or rs9296158.

## Discussion

This is the first study examining the association between severe childhood abuse and functional connectivity of brain networks during sustained attention in medication-naïve, drug-free young people. Furthermore, the exploration of GxE effects on maltreatment-related connectivity abnormalities is novel. Behaviorally, maltreated individuals exhibited increased intrasubject variability, premature and omission errors, the main attention measure of the task. Abused participants relative to healthy controls exhibited significantly reduced functional connectivity between left DLPFC and left IPL, supramarginal gyrus, IFC, post- and precentral gyri and between left IPL and bilateral DLPFC and rmPFC during sustained attention. No correlations were observed between functional connectivity deficits and abuse onset, duration or severity. Abuse-related deficits in left hemispheric fronto-parietal connectivity were moderated by FKBP5 Genotype, specifically SNP rs3800373.

Young people with a history of severe childhood abuse showed reduced connectivity relative to healthy controls during the most challenging attention condition in predominantly left hemispheric dorsal and ventral fronto-parietal networks that are known to be important for sustained attention. The fact that extremely similar prefrontal-parietal networks were shown to be affected in connectivity analyses of both the left DLPFC and the left IPL seed regions corroborates and reinforces the finding of fronto-parietal network dysfunctions during attention. DLPFC (BA 46) plays a crucial role in top-down attention and is activated during visuospatial information processing and orienting of attention [58, 74, 79], rmPFC (BA 10) has been implicated in attention during prospective memory paradigms, i.e. carrying out an intended action after a delay [84, 85] and IPL is also a key region in the control of sustained attention [77, 86, 87]. DLPFC, rmPFC and IPL have been associated with sustained attention during this particular task version [53, 54]. The well-established role of fronto-parietal networks in sustained attention [88, 89] is consistent with the theory that decreased connectivity of these networks in maltreated individuals contributes to behavioural attention deficits observed in the current study and the neuropsychological literature in the form of increased omission errors [7, 9–11, 15]. The lack of a correlation between attention measures and connectivity findings in the current study may be related to relatively low power for correlation analyses.

The structure and function of the prefrontal cortices, including DLPFC, rmPFC and IFC, are consistently reported to be affected by childhood maltreatment [2, 3, 24], and there is also some evidence for alterations in parietal regions [4]. Our previous fMRI findings using the same task and subjects found abnormally reduced activation in the abused group in typical dorsal and ventral attention networks including left DLPFC, IFC and temporal regions [18]. The finding of diminished functional connectivity for maltreated adolescents, relative to healthy controls, between the prefrontal cortex and parietal lobe extends these previous findings of hypoactivity in DLPFC and IFC to the network level by showing that the functional communication between these regions is disturbed and not just their activation.

The findings also extend previous structural connectivity findings that demonstrate that adolescents exposed to childhood maltreatment have reduced density of bilateral superior longitudinal fasciculi, white-matter tracts that connect prefrontal areas, including the DLPFC, to parietal regions [90]. Interestingly, recent findings, combining diffusion imaging MRI data with magnetoencephalography, implicate the medial branch of the superior longitudinal fasciculus, in top-down control of neuronal synchronisation associated with selective attention [91]. The human brain is plastic and is continually modified by experience across development. Given that prefrontal and parietal regions are among the latest brain regions to develop structurally [78] and functionally [92], developing well into mid-adulthood, their protracted development may render fronto-parietal networks more susceptible to impairment following childhood adversity.

Our preliminary GxE findings in fronto-parietal connectivity are intriguing as they suggest that connectivity deficits in these stress-susceptible error processing brain networks were influenced by the abuse experience and possibly exacerbated in the presence of the risky C-allele of the rs-3800373 SNP of the FKBP5 gene, an effect which seemed only to be present in individuals homozygous for the C-allele. It should, however, be noted that these results are merely exploratory as subject numbers are too small to make any conclusions regarding GxE but it does highlight a possible relationship that warrants further investigation. C-allele carriers of rs-3800373 exposed to childhood maltreatment have been shown to demonstrate increased risk of PTSD [46], limbic irritability, depression, dissociation [47], suicide attempts [93], aggression and violence [94]. No group-by-5-HTTLPR nor MAOA effects were observed suggesting that the specific fronto-parietal functional connectivity deficits observed during sustained attention are not modulated by 5-HTTLPR or MAOA genotype.

Among the strengths of the current study is that all participants were medication-naïve, drug-free and the abuse experience was carefully assessed and corroborated by social service records. It is unclear to what extent pubertal development, malnutrition, prenatal drug exposure and presence of current life stressors may have influenced the findings. The SES measure used is limited, as it does not provide information on parents’ income and education; however, youth often have difficulties in reporting this information [95]. The cross-sectional nature of the study is a further limitation. As the affected fronto-parietal networks develop well into mid-adulthood [78, 92] the true impact of childhood maltreatment on functional connectivity may not be revealed in this adolescent sample. Although we recruited participants exposed to childhood physical abuse, it is unrealistic to separate physical abuse from typically co-occurring emotional abuse and neglect [50, 51]; hence, our abuse group had experienced emotional abuse and neglect as well. An important future direction for research is to investigate the way in which sexual abuse affects functional connectivity during sustained attention to elucidate potential differences in the way distinct abuse types affect neuronal networks. Another limitation is the inclusion of mixed genders as maltreatment may affect the genders differently [96].

## Conclusions

In summary, using medication-naïve, drug-free, carefully assessed age-matched groups of young people exposed to severe childhood maltreatment and healthy controls, we found that abused participants had reduced functional connectivity of primarily left hemispheric fronto-parietal networks, including DLPFC and IPL, during sustained attention. Furthermore connectivity deficits were moderated by FKBP5 genotype. Hence, in response to an abusive early environment maltreated individuals may develop a reduction in communication between brain regions involved in sustained attention resulting in attention deficits. These findings represent a first step towards the delineation of abuse-related neurofunctional connectivity abnormalities, which hopefully will facilitate the development of specific treatment strategies for victims of childhood maltreatment.

## Supporting information

S1 Fig Schematic representation of the sustained attention task. Subjects are required to press a right-hand button as soon as they see a timer appear on the screen counting seconds. The counter appears after either predictable short delays of 0.5s in blocks of 3–5 stimuli, or after unpredictable long delays of 2s, 5s or 8s, pseudorandomly interspersed into the blocks of 0.5s delays. The long second delays have a progressively higher load on sustained attention than the short 0.5s delays that are typically anticipated and have a higher load on sensorimotor synchronization.
(TIF)

S2 Fig Within group functional connectivity for the 10 seed regions for the 8s delay sustained attention condition. The threshold is P < 0.05 FWE corrected. The right of the image corresponds to the right side of the brain. L = left, R = right, ACC = anterior cingulate cortex, IFC = inferior frontal cortex, SMA = supplementary motor area.
(TIF)

S1 Table Exploratory IQ ANCOVA analysis. Regions demonstrating differential functional connectivity with the left dorsolateral prefrontal cortex and left inferior parietal lobe seed regions during the 8s delay versus 0.5s implicit baseline condition for 21 young people exposed to severe childhood abuse and 27 healthy controls, when covarying for IQ. P-value is <0.05 FWER corrected.
(DOCX)
