# Supplementary figures and images for: Reduced functional connectivity of fronto-parietal sustained attention networks in severe childhood abuse
Source: PLoS One. 2017 Nov 30;12(11):e0188744. doi: 10.1371/journal.pone.0188744 (PMC5708742; doi:10.1371/journal.pone.0188744)

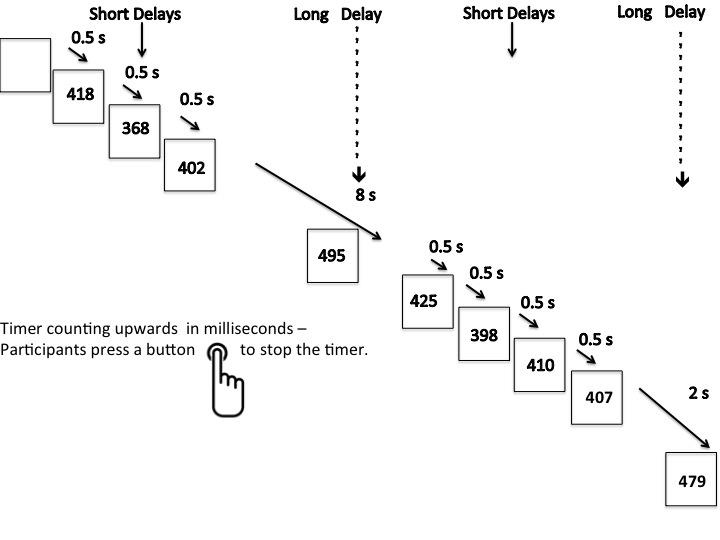

Supplement: S1 Fig — Subjects are required to press a right-hand button as soon as they see a timer appear on the screen counting seconds. The counter appears after either predictable short delays of 0.5s in blocks of 3–5 stimuli, or after unpredictable long delays of 2s, 5s or 8s, pseudorandomly interspersed into the blocks of 0.5s delays. The long second delays have a progressively higher load on sustained attention than the short 0.5s delays that are typically anticipated and have a higher load on sensorimotor synchronization. (TIF) [file pone.0188744.s001.tif]

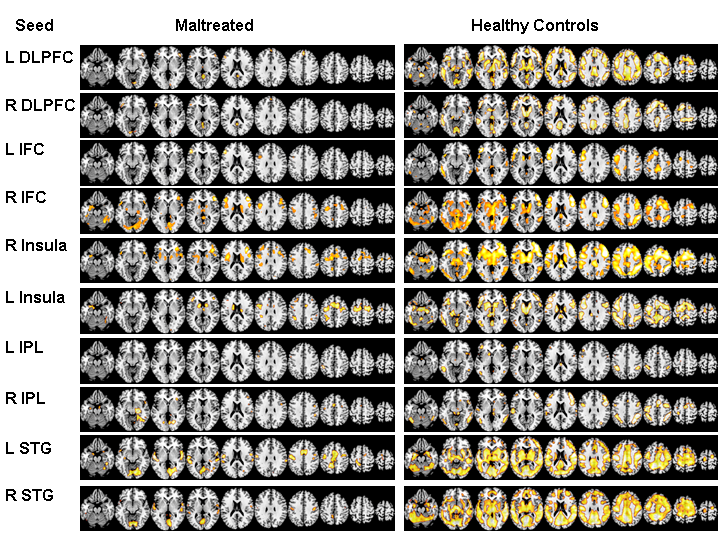

Supplement: S2 Fig — The threshold is P < 0.05 FWE corrected. The right of the image corresponds to the right side of the brain. L = left, R = right, ACC = anterior cingulate cortex, IFC = inferior frontal cortex, SMA = supplementary motor area. (TIF) [file pone.0188744.s002.tif]
