# Supplementary material for: Reduced functional connectivity of fronto-parietal sustained attention networks in severe childhood abuse
Source: PLoS One. 2017 Nov 30;12(11):e0188744. doi: 10.1371/journal.pone.0188744 (PMC5708742; doi:10.1371/journal.pone.0188744)
Supplement: S1 Table — Regions demonstrating differential functional connectivity with the left dorsolateral prefrontal cortex and left inferior parietal lobe seed regions during the 8s delay versus 0.5s implicit baseline condition for 21 young people exposed to severe childhood abuse and 27 healthy controls, when covarying for IQ. P-value is <0.05 FWER corrected. (DOCX) [file pone.0188744.s003.docx]

|  |  | **Cluster Level** | | **Peak** | **Voxel Level** |
| --- | --- | --- | --- | --- | --- |
| **Seed Region** | **Comparison and Brain Regions** | **No. of Voxels** | ***p (corr)*** | **MNI Coordinates^b^** | **Z** |
| **L DLPFC** | **Physically Maltreated < Healthy Controls** | | | | |
|  | Left inferior parietal lobe, inferior frontal, postcentral gyrus (BA 40/44/3) | 344 | 0.032 | -58,-40,28 | 4.03 |
| **L IPL** | **Physically Maltreated < Healthy Controls** | | | | |
|  | Bilateral dorsolateral and rostromedial prefrontal cortex (BA 46/10) | 271 | 0.041 | -10,58,-4 | 3.96 |
